# CSF Cytokines in Aging, Multiple Sclerosis, and Dementia

**DOI:** 10.3389/fimmu.2019.00480

**Published:** 2019-03-15

**Authors:** William T. Hu, Jennifer Christina Howell, Tugba Ozturk, Umesh Gangishetti, Alexander L. Kollhoff, Jaime M. Hatcher-Martin, Albert M. Anderson, William R. Tyor

**Affiliations:** ^1^Department of Neurology, Emory University, Atlanta, GA, United States; ^2^Center for Neurodegenerative Disease, Emory University, Atlanta, GA, United States; ^3^Alzheimer's Disease Research Center, Emory University, Atlanta, GA, United States; ^4^Jean and Paul Amos Parkinson's Disease and Movement Disorders Program, Emory University, Atlanta, GA, United States; ^5^Department of Internal Medicine, Emory University, Atlanta, GA, United States; ^6^Atlanta VA Medical Center, Decatur, GA, United States

**Keywords:** inflammaging, neuroinflammation and neurodegeneration, IP10, IL10, IL8, Alzheimer's disease, Parkinsons disease (PD), dementia with Lewy bodies (DLB)

## Abstract

Inflammation is a common process involved in aging, multiple sclerosis (MS), and age-related neurodegenerative disorders such as Alzheimer's disease (AD) and Parkinson's disease (PD), but there is limited evidence for the effects of aging on inflammation in the central nervous system. We collected cerebrospinal fluid (CSF) from 105 healthy control subjects representing a wide age range (23–86), and analyzed levels of cytokines associated innate immunity (TNF-α) and different T-helper subtypes: interferon–gamma induced protein 10 (IP-10) for Th1, interleukin-10 (IL-10) for Th2, and interleukin 8 (IL-8/CXCL8) for Th17. We show that CSF levels of TNF-α, IP-10, and IL-8 all increased linearly with age, but levels of IL-10 demonstrated a U-shaped relationship with age. We further found greater age-related increases in TNF-α, IL-10, and IL-8 relative to increases in IP-10 levels, consistent with a shift from Th1 to other inflammatory phenotypes. Finally, when we analyzed the same four cytokines in people with neurological disorders, we found that MS and AD, but not PD or dementia with Lewy bodies, further accentuated the age-related shift from Th1- to non-Th1-related cytokines. We propose that CSF cytokine levels represent powerful surrogates of brain inflammation and aging, and some, but not all, neurological disorders accelerate the shift away from Th1 phenotypes.

## Introduction

Neuro-inflammation is increasingly implicated in age-related neurodegenerative diseases such as Alzheimer's disease (AD) and Parkinson's disease (PD) (1, 2). Following examples in multiple sclerosis (MS) and other demyelinating conditions, many recent human neurodegenerative studies have analyzed soluble cytokine levels in the blood or cerebrospinal fluid (CSF) to generate hypotheses testable in other cohorts or animal models (3–5). However, factors such as age can themselves bias the immune system and therefore cytokine levels, including aging-associated pro-inflammatory bias and a shift from Th1 to Th2/innate immunity dominance (6, 7). These patterns, usually sterile (free of symptomatic infection), independent of organ- or system-based disease, and involving the innate and adaptive immune systems, have been together referred to as inflamm-aging (8, 9). Potential drivers for inflamm-aging include persistent low-grade viral and bacterial infection, changing gut microbiome (10), and clearance of accumulating self-proteins. Because of inflamm-aging, it is not always straightforward to distinguish between the effects of disease and non-disease factors on absolute cytokine levels. A thorough evaluation of disease-associated cytokine alterations thus requires an adequate number of healthy subjects across a wide age range to detect not only cytokine level differences but also multi-cytokine profiles, which inform about disease specific changes that also reflect complex immune pathways.

Because recruiting healthy subjects to undergo CSF collection can be difficult, multiple smaller cohorts may need to be combined to generate a larger cohort. This process is not without its own challenges, including the need for standardized CSF collection across multiple research centers with disease-specific protocols (11, 12), reproducible cytokine quantitation, and data-sharing. Over the past 4 years, we have recruited 105 healthy control (HC) subjects over a wide age-range (22–86) to participate in multiple disease-oriented studies using a modified but standardized Alzheimer's Disease Neuro-Imaging (ADNI) protocol (12). This cohort provides a unique opportunity to simultaneously interrogate CSF cytokines from different functional pathways across seven decades of life. Here we measured four cytokines associated with innate immunity and helper T cell subtypes (Th1, Th2, Th17) in these HC subjects, and present a new framework to assess imbalance between cytokine pairs in central nervous system (CNS) disorders including MS, AD, PD, and dementia with Lewy bodies (DLB).

## Materials and Methods

### Standard Protocol Approvals and Patient Consents

This study was carried out in accordance to US Code of Federal Regulations Title 45 Part 46 Protection of Human Subjects, and Emory University and Emory School of Medicine policies. The protocols were approved by the Emory University Institutional Review Board. Banked CSF samples were used for this study, and all subjects had previously given written informed consent according to the Declaration of Helsinki for long-term sample storage and future analysis.

### Subject Characterization

Older HC and AD subjects (*n* = 52, median age 69, range 48–89) were recruited during a previous study on CSF and MRI biomarkers of aging and dementia in Caucasian and African Americans (13). Younger HC subjects were recruited from the Emory Cognitive Neurology Clinic, Emory Alzheimer's Disease Research Center, and Emory University in an on-going study of pre-symptomatic carriers for dominantly-inherited frontotemporal lobar degeneration, and a separate study examining CSF biomarkers of HIV. All HC subjects underwent detailed neuropsychological testing (14) to confirm normal cognition. Older and younger HC subjects were then combined to form a continuum (median age 60; range 22–105), with 52/105 (50%) HC subjects younger than 60 years of age. MS (*n* = 18, median age 48, range 28–74), PD (*n* = 37, median age 69, range 41–81), and DLB (*n* = 23, median age 68, range 47–80) were recruited from the Emory Neuroimmunology, Movement Disorders, and Cognitive Neurology Clinics. Because recruitment was completed prior to the latest revisions in diagnostic criteria for MS and DLB in 2017, all MS patients were diagnosed according to the 2010 revised McDonald criteria (15), and all DLB patients were diagnosed according to the 2005 McKeith criteria (16). PD patients had clinical features and findings consistent with the Movement Disorders Society Parkinson's disease criteria (17).

### CSF Collection

CSF samples were all previously collected using a modified Alzheimer's Disease Neuroimaging Initiative protocol at Emory University (18). Briefly, CSF was collected into 15 mL polypropylene tubes via a 24-gauge atraumatic needle and syringe aspiration without overnight fasting. CSF in polypropylene tubes was immediately inverted several times, aliquotted (500 μL), labeled, and frozen at −80°C until analysis. CSF samples from 13 young HC subjects (mean age 37.0, range 23–54) were centrifuged at 2,500 rpm after collection before freezing. We previously carried out a prospective experiment centrifuging in this condition half of freshly collected CSF in 16 subjects, and compared levels of eight CSF cytokines (including the four included in the current study) in the supernatant with levels from the uncentrifuged halves. We showed that centrifugation did not influence measured cytokine levels (19), and these samples can be analyzed together. We have also determined the stability of each analyte through freeze-thawing following a pre-established protocol such that the measured levels from frozen samples most closely reflect *in vivo* levels (18).

### CSF Cytokine Analysis

Four inflammatory proteins were selected for their preferential association with innate immunity or different immune cell populations, including tumor necrosis alpha (TNF-α) (20), Th-2 related interleukin 10 (IL-10) (21), and Th17-related interleukin 8 (IL-8/CXCL8) (22). Levels of Th1-associated interferon gamma were not consistently detectable, and a downstream marker interferon gamma-induced protein (IP-10/CXCL10, Th1) (23) was used instead as a surrogate. Assays were performed in a Luminex 200 platform using the Merck-Milliplex MAP Human Cytokine Panel (HCYTOMAG-60K, Merck-Millipore, Burlington, MA) following the manufacturer's protocol except two 100 μL aliquots of CSF were used for duplicates instead of what was stated in the protocol. IL-9 is also associated with Th17 pathways but its CSF alterations are challenging to interpret because of influence from race and potentially other factors (Wharton and Hu, unpublished data). Analysis involving IL-9 was thus deferred here. IL-6 was also not measured because its CSF levels were found to be normal in multiple previous studies including in aging, AD (5, 24, 25), and MS (26–29), potentially confounded by the large inter-individual variability (30). Intermediate precision achieved in our laboratory using thirteen biological replicates (different aliquots from the same lumbar puncture for thirteen subjects) over 9 weeks was 4.8% for IP-10, 9.4% for TNF-α, 16.5% for IL-10, and 12.0% for IL-8.

### Statistical Analysis

HC subjects were analyzed with age as a continuous variable or according to categories each spanning two decades of life: 20–39, 40–59, and 60–79. Six subjects older than 80 (range 81–86) were combined with the last group to form the category of ≥60. For basic demographic comparison, Chi-squared tests and analysis of variance were used to determine categorical and continuous baseline variables across the five diagnostic categories (HC, MS, AD, PD, DLB). CSF IP-10 levels did not have a normal distribution, and were thus log_10_-transformed. Analysis of co-variance (ANCOVA) was used to analyze the main effect of diagnosis on CSF cytokine levels, adjusting for age and sex. Pearson's correlational analysis was used to determine the association between cytokines. Regression analysis was used to determine the impact of inflamm-aging in HC subjects with log_10_-transformed IP-10, age, and gender, and the interaction between age and log_10_(IP-10) as variables.

To determine impact of neurological disorders on inflamm-aging, levels of log_10_(IP-10), TNF-α, IL-10, and IL-8 were Z-transformed to adjust for absolute level and distribution differences between the cytokines. Specifically, mean and standard deviation values were calculated for each cytokine from all HC subjects, and all subjects' cytokine levels were then converted to Z-scores. Regression analysis was then used to determine the impact of disease on inflamm-aging, with log_10_(IP-10), age, gender, age X log_10_(IP-10), and diagnosis X log_10_(IP-10) as variables.

## Results

**Figure 1:**
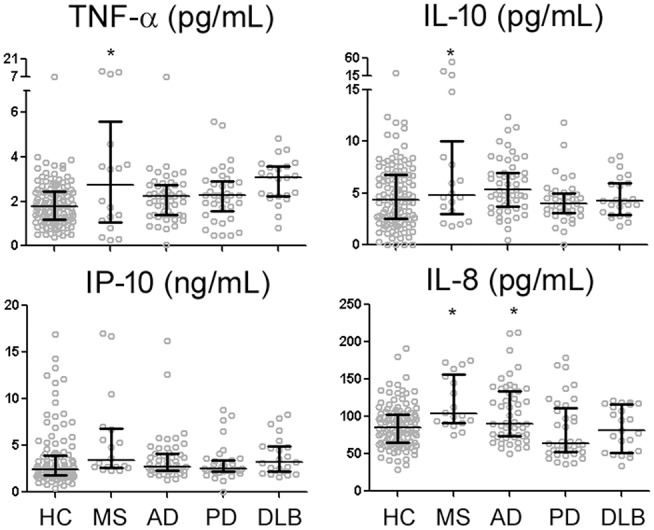
CSF levels of four reliably-measured cytokines in healthy control subjects (HC), multiple sclerosis (MS), Alzheimer's disease (AD), Parkinson's disease (PD), and dementia with Lewy bodies (DLB). ^*^p < 0.05 compared to HC.

Among 105 HC subjects, CSF levels of TNF-α, IL-10, and IP-10 all strongly correlated with one another (R range of 0.354–0.449, *p* < 0.001), while IL-8 only correlated with IP-10 levels (*R* = 0.469, *p* < 0.001). Univariate analysis showed higher levels of TNF-α, IP-10, IL-10, and IL-8 in MS than HC (Figure 1). In contrast, univariate analysis identified AD as the only non-MS disorder associated with higher levels of a single cytokine (IL-8) than HC (Figure 1). Regression analysis including HC and MS patients taking into account age, gender, MS diagnosis, and MS treatment showed MS treatment to be associated with relative decrease in IL-10 levels (*p* = 0.014).

**Figure 2:**
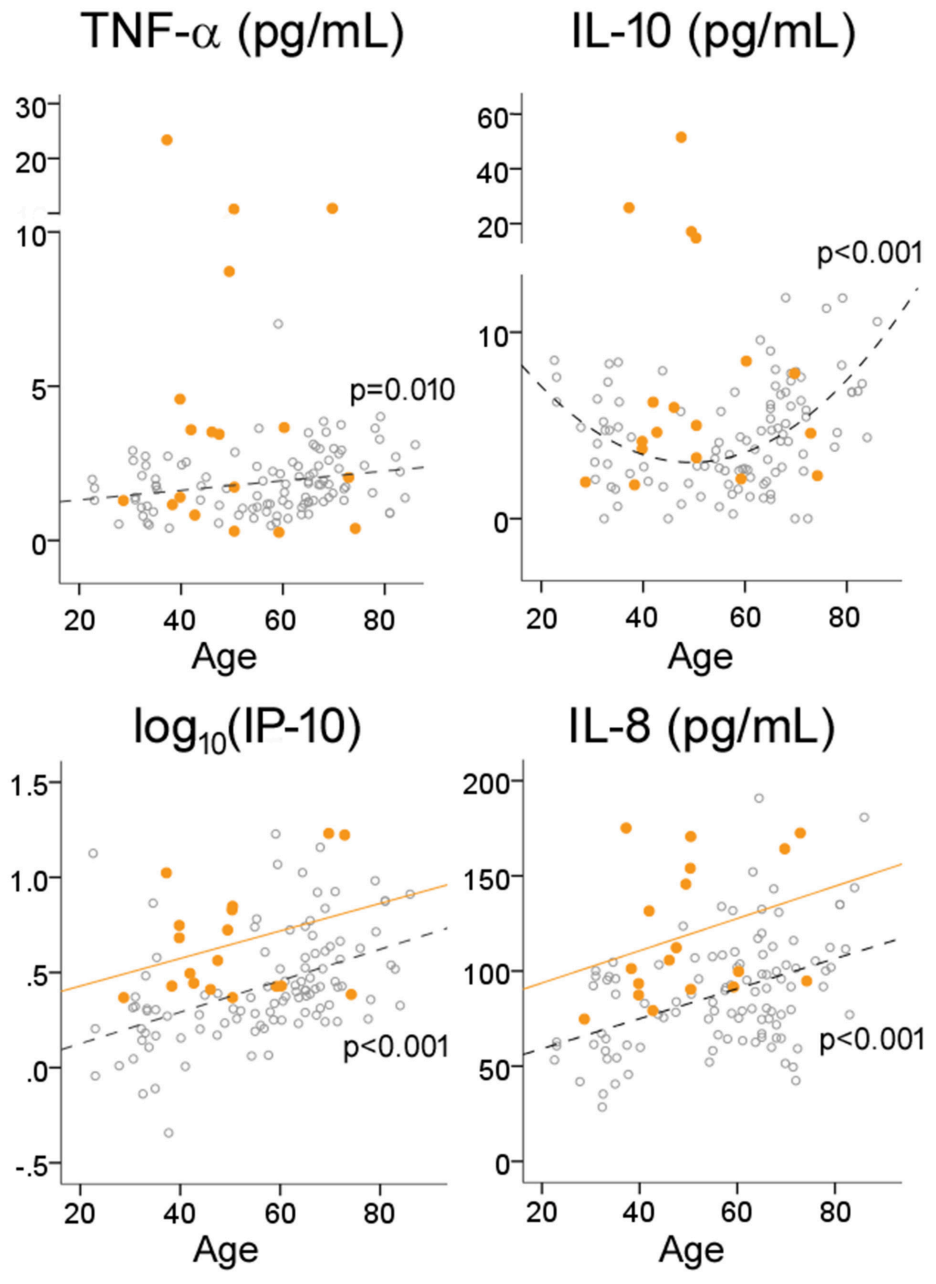
Effects of age on cytokine levels in HC and MS. Open circles and dotted lines (with associated *p*-values) represent individual values and best fit lines for HC subjects; orange circles and orange lines represent individual values and best fit lines for MS subjects.

Because inflamm-aging can potentially complicate the interpretation of group-level comparisons, we next explored the effects of age on CSF cytokine levels in HC subjects using regression analysis. Consistent with a pro-inflammatory bias, TNF-α (*R* = 0.250, *p* = 0.010), IP-10 (*R* = 0.453 for log_10_-transformed values, *p* < 0.001) and IL-8 (*R* = 0.439, *p* < 0.001) levels all increased with age (Table 1, Figure 2) while controlling for gender. Interestingly, instead of a linear or step-wise increase, we observed a trend of lower CSF IL-10 levels in the middle-aged group (40–59) than the older and younger groups (Table 1). This led us to model this U-shaped relationship between age and IL-10 levels using a quadratic equation (Figure 2, *R* = 0.495, *p* < 0.001), with the inflection point (lowest levels) at 50 years of age. ANCOVA showed that MS was associated with greater IP-10 [*F*_(2, 123)_ = 14.537, *p* < 0.001], TNF-α [*F* = 19.321, *p* < 0.001], IL-10 [*F* = 18.921, *p* < 0.001], and IL-8 [*F* = 23.358, *p* < 0.001] levels (Figure 2), but only IP-10 and IL-8 increased with age in MS (*p* < 0.001 for both).

**Table 1:** Basic demographic and CSF cytokine levels of people with healthy control (HC) with normal cognition according to age group as well as multiple sclerosis (MS).

	**MS (*n* = 18)**	**NC, 20–39 year (*n* = 23)**	**NC, 40–59 year (*n* = 29)**	**NC, ≥60 year (*n* = 53)**
Male	4 (22%)	16 (70%)	11 (38%)	24 (45%)
Age (SD), year	50.0 (12.8)	31.9 (4.2)	52.8 (5.8)	69.5 (6.6)
Disease duration (SD), yr	11.0 (12.9)	–	–	–
MS Type				
RRMS	15			
PPMS	2			
SPMS	1			
Current MS therapy	8			
Interferon β-1a	2			
Glatiramer acetate	2			
Fingolimod	2			
Dimethyl fumarate	1			
Natalizumab	1			
TNF-α (SD), pg/mL	4.55 (5.78)^*^	1.51 (0.76)	1.75 (1.28)	2.11 (0.89)
IP-10 (SD), pg/mL	5.61 (4.64)^**^	2.46 (2.71)	3.09 (3.38)	4.07 (2.65)
IL-10 (SD), pg/mL	9.51 (12.20)^*^	4.57 (2.48)	2.82 (1.87)	5.30 (2.93)
IL-8 (SD), pg/mL	119.1 (35.2)^*^	67.5 (22.9)^†^	88.4 (21.3)	96.4 (31.5)

*RRMS, relapsing-remitting MS; PPMS, primary progressive MS; SPMS, secondary progressive MS*.

* *Higher in MS than other groups*.

** *Higher in MS than 22–39 and 40–59 year old groups*.

† *Lower in the youngest group than the other two*.

Beyond paralleled increases in cytokine levels beyond the age of 50, we further analyzed if age modified the relationship between Th1-associated IP-10 and other cytokines by examining the interaction between age and IP-10. This showed that, not only is there an age-associated increase in these CSF cytokines, age further accentuated the positive correlation between IP-10 and TNF-α, IL-10, and IL-8 levels (*p* < 0.001 for the interaction terms, Table 2) after controlling for gender. These findings suggest relatively greater increases in these three cytokines for each standard unit of age-associated increase in IP-10, in keeping with a consistent phenotype switch from Th1 to innate immunity (31) and Th2 (7) phenotypes.

**Table 2:** Age modifies the relationship between IP-10 and other cytokines in HC (coefficients and *p*-values are shown).

	**TNF-α**	**IL-10**	**IL-8**
Age, year	−0.007 (*p* = 0.357)	−0.052 (*p* = 0.053)	−6.259 (*p* = 0.207)
Male gender	0.133 (*p* = 0.456)	0.955 (*p* = 0.047)	−6.838 (*p* = 0.161)
log_10_(IP-10)	0.287 (*p =* 0.780)	−3.985 (*p =* 0.146)	−4.000 (*p =* 0.888)
Age X log_10_ (IP-10)	0.023 (*p* < 0.001)	0.146 (*p* = 0.003)	0.759 (*p* < 0.001)

**Figure 3:**
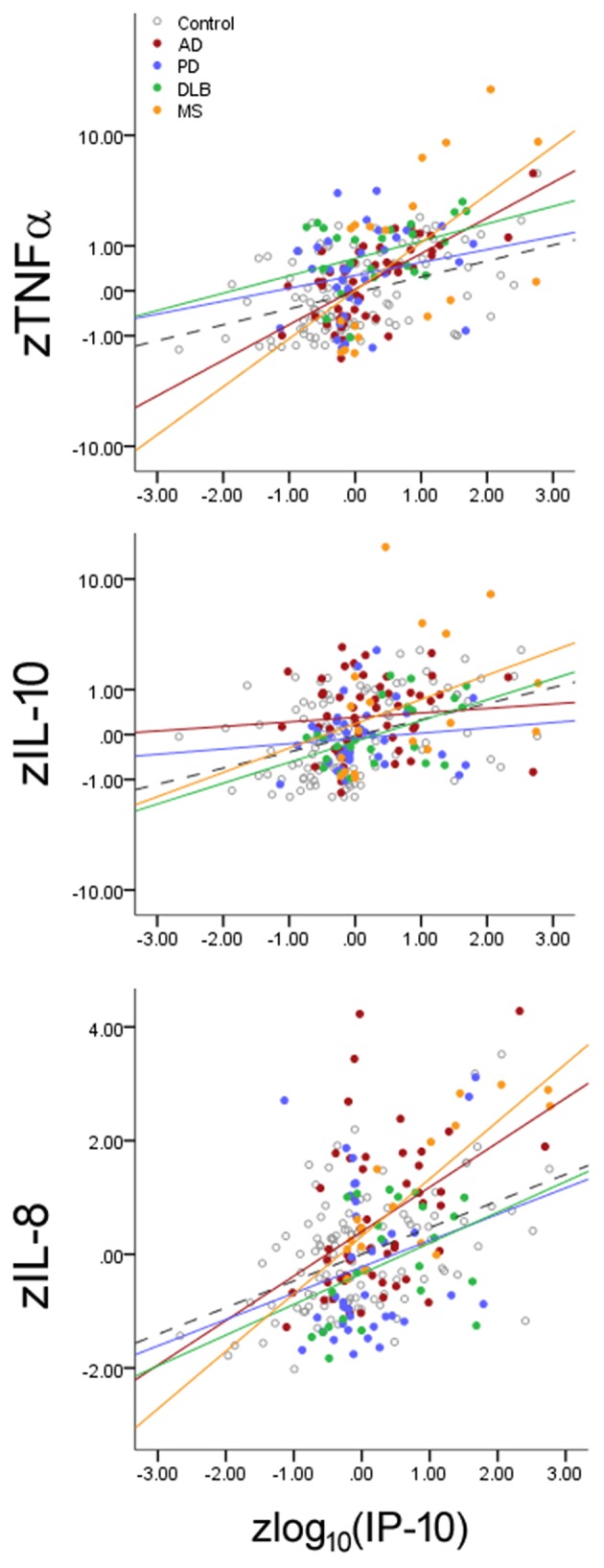
Effects of neurological diseases on the relationship between Th1-associated IP-10 and cytokines associated with innate immunity (TNF-α), Th2 (IL-10), and Th17 (IL-8). Cytokine levels were normalized according to HC mean and standard deviation values for illustrative purposes. Accounting for inflamm-aging showed that MS and AD enhanced the relationship between these cytokines, while PD and DLB changed the absolute cytokine levels without altering the relationships. Dotted lines represent best fit line for HC subjects.

While inflammatory changes are either pathogenic (e.g., MS) or commonly observed in neurodegenerative disorders (e.g., AD, PD), it is not clear if each neurological disorder can specifically alter the balance between cytokines linked to inflamm-aging. We thus analyzed if IP-10 levels interacted with disease status in addition to its interaction with age (Figure 3) by examining if each disease alters the slope linking IP-10 and the three other cytokines. We found that, for each standard deviation increase in IP-10 beyond the age-associated effect on IP-10, both MS (*p* < 0.001) and AD (*p* = 0.026) were associated with greater change in TNF-α than HC, AD was associated with greater change in IL-10 levels (*p* = 0.018) than HC, and MS was associated with greater change in IL-8 levels (*p* = 0.013) than HC. Importantly, these interactions were independent of the age-associated shift. While PD and DLB were associated with greater TNF-α levels and lower IL-8 levels than NC, these disorders did not modify the inflamm-aging relationships between IP-10 and other cytokines. Combining subjects with PD and DLB as Lewy body disease did not change the outcomes.

## Discussion

Inflamm-aging is known to occur in the blood (32, 33) and bone marrow (34), but only limited evidence exists in the human CNS (35, 36). Here we show in a cross-sectional cohort spanning seven decades of life that CSF levels of cytokines associated with innate immunity, Th1, Th2, and Th17 all increased with age. We further show that these cytokines did not increase according to age at the same rates, and aging was associated with greater relative increases in cytokines levels linked to innate immunity, Th2, and Th17 than Th1. Finally, we show that MS and AD further accentuate the age-associated phenotype shift from Th1 to non-Th1 pathways independent of inflamm-aging. Altogether, these CSF findings provide evidence for inflamm-aging in the CNS, and put forth the hypothesis that some–but not all–neurological disorders mimic key features of inflamm-aging.

Inflamm-aging is thought to result from chronic unresolved infection and immunosenesence among other factors (37). There is a general paucity of evidence for inflamm-aging in the human CNS due to limited availability of *normal* post-mortem brain tissue across the life span (36, 38, 39). Using brains from 57 neuropathologically and cognitively normal people from seven brain banks, an expression analysis showed aging and AD to each upregulate genes associated the complement pathway and the toll-like receptors (39). They also identified brain IL-10 as a cytokine implicated in inflamm-aging, in keeping with our findings of age- and AD-associated increase in IL-10 relative to IP-10 elevations. However, a difference in brain IL-10 expression was not reported by a subsequent RNA-Seq study examining 79 subjects when cases were analyzed according to age (under 60 or over 60) (36), possibly due to the U-shaped distribution of CSF IL-10 levels according to age. This age-dependent U-shaped curve can also potentially account for inconsistent findings of normal (28, 40, 41) or elevated (29, 42). CSF IL-10 levels in MS patients from cross-sectional studies. At the same time, the aging-associated trough for CSF IL-10 levels in the fifth decade may identify a window during which the brain is susceptible to immune-mediated processes. Because the peak age at onset for MS precedes this natural IL-10 decline by one to two decades, it is conceivable that patients with MS may experience this IL-10 trough–and the associated unopposed increase in TNF-α, IP-10, and IL-8–earlier in life. Indeed, age at onset has prognostic roles in initial phenotype, progression, and disability (43–45), and the CSF cytokine milieu at onset may better represent biological age with respects to neuroinflammation than chronological age. While definitive testing of this hypothesis would require pre-symptomatic identification of MS patients, healthy young adults at increased risks for MS through family history or a GWAS-based polygenic risk score (46) may determine candidate genes associated with premature IL-10 decline.

Increased CSF TNF-α, IP-10, and IL-8 levels have been previously reported in untreated and treated MS (29, 47–51). A treatment-associated decrease in IL-10 levels in MS may seem contradictory at first especially if premature IL-10 decline may be a risk factor for MS. Previous studies have shown coupled increase of CSF TNF-α and IL-10 during MS flare-ups (52) as well as their coupled normalization after interferon-β or natalizumab treatment (53, 54). Therefore, lower IL-10 levels in treated vs. untreated MS patients likely reflect generalized reduction in immune activation, and MS-treatment does not attenuate the bias toward IL-10 from IP-10. It remains to be determined whether individual cytokine levels or the relative balance between cytokines better predict disease progression or response to therapy.

The exact mechanisms underlying inflamm-aging are not clear. Relevant to the CNS, aging is associated with dendritic remodeling (55), reduced CSF outflow (56), increasing risks of tau-related pathology (57), premature immunosenescense (58), and often white matter hyperintensity associated with cerebrovascular disease on MRI. While we cannot clearly distinguish between CSF cytokine changes due to chronological aging, immunosenescence, and cumulative subclinical injury common in aging (e.g., ischemia, hypoxia), our work–to the best of our knowledge–provides the first CSF-based evidence for CNS inflamm-aging. Major strengths of the current study include the pooling of subjects with HC and neurological disorders based on standardized collection protocols across multiple sites, and the joint analysis of cytokine levels with age-adjusted balance between cytokines. At the expense of scope, we restricted our analysis to four CSF analytes for their readily detectable levels (e.g., CSF IFN-γ and IL-4 are not easily detected in most CSF samples), measurement accuracy (e.g., some commercial assays fail to validate when non-kit protein standards are used), high intermediate precision (reproducibility between assays run on different days), stability during processing (e.g., freeze-thawing), and invariance across racial groups. Omitting cytokine measures that fall short on specificity or reproducibility allowed for more consistent statistical analysis to address inflamm-aging and to detect linear as well as the U-shaped trends. It is important to note that the cross-sectional nature of our study is suggestive–not confirmatory–of an age-related change over time. An independent validation cohort and longitudinal analysis within the same individuals are necessary to confirm these findings. We also did not quantify different cell types in the CSF, and immunophenotyping will be necessary to test whether cytokine level changes are due to cell type switch, hyperactivity, or both. Because of our focus on CNS inflamm-aging, we did not measure plasma cytokines because we have previously found little correlation between plasma and CSF cytokine levels in HC or people with neurological diseases. In keeping with this, prior studies reported dissociated patterns of plasma and CSF cytokine alterations in MS, but a comparative study of peripheral and CNS inflamm-aging may bring about additional insight. Nevertheless, we present a new framework for simultaneously evaluating CSF cytokines related to inflamm-aging and neurological disorders that goes beyond up- or down-regulation, and extends the Th1 to non-Th1 shift associated with aging to AD.

## Data Availability

The raw data supporting the conclusions of this manuscript will be made available by the authors, without undue reservation, to any qualified researcher.

## Author Contributions

WH, JH, and UG contributed to conception and design of the study. WH, JH, TO, UG, AK, JH-M, AA, and WT organized the dataset. WH performed the statistical analysis. WH and JH wrote sections of the manuscript. TO, UG, JH-M, AA, and WT contributed to the revision of the manuscript. All authors read and approved the submitted version.

### Conflict of Interest Statement

WH has a patent on the use of CSF biomarkers to diagnose FTLD-TDP (US 9,618,522); has received research support from Avid Radiopharmaceuticals (Philadelphia, PA) and Fujirebio US (Malvern, PA); serves as a consultant to AARP, Inc, Locks Law Firm, Interpleader Law, and Roche Diagnostics USA, ViveBio LLC; has received travel support from Hoffman-LaRoche and Abbvie. The funder played no role in the study design, the collection, analysis or interpretation of data, the writing of this paper or the decision to submit it for publication. The remaining authors declare that the research was conducted in the absence of any commercial or financial relationships that could be construed as a potential conflict of interest.
